# Young women's decisions to accept chlamydia screening: influences of stigma and doctor-patient interactions

**DOI:** 10.1186/1471-2458-10-425

**Published:** 2010-07-19

**Authors:** Myles Balfe, Ruairi Brugha, Diarmuid O'Donovan, Emer O'Connell, Deirdre Vaughan

**Affiliations:** 1 Department of Epidemiology and Public Health, Royal College of Surgeons in Ireland, Dublin, Ireland; 2 Department of Health Promotion, National University of Ireland Galway, Galway, Ireland

## Abstract

**Background:**

An understanding of the factors that encourage young women to accept, and discourage them from accepting, STI (sexually transmitted infection) testing is needed to underpin opportunistic screening programs for the STI *Chlamydia trachomatis *(opportunistic screening involves healthcare professionals offering chlamydia tests to people while they are attending health services for reasons that are usually unrelated to their sexual health). We conducted a qualitative study to identify and explore: how young women would feel about being offered opportunistic tests for chlamydia?; how young women would like to be offered screening, and who they wanted to be offered screening by?; and what factors would influence young women's partner notification preferences for chlamydia (who they would notify in the event of a positive diagnosis of chlamydia, how they would want to do this).

**Methods:**

Semi-structured interviews with 35 young women between eighteen and twenty nine years of age. The study was conducted in the Dublin and Galway regions of the Republic of Ireland. Young adults were recruited from General Practice (GP) practices, Third Level College health services, Family Planning clinics and specialist STI treatment services.

**Results:**

Respondents were worried that their identities would become stigmatised if they accepted screening. Younger respondents and those from lower socio-economic backgrounds had the greatest stigma-related concerns. Most respondents indicated that they would accept screening if it was offered to them, however; accepting screening was seen as a correct, responsible action to engage in. Respondents wanted to be offered screening by younger female healthcare professionals. Respondents were willing to inform their current partners about positive chlamydia diagnoses, but were more ambivalent about informing their previous partners.

**Conclusions:**

If an effort is not put into reducing young women's stigma-related concerns the population coverage of Chlamydia screening might be reduced.

## Background

*Chlamydia trachomatis *is a sexually transmitted infection (STI) that, if left untreated, can damage women's fertility [1]. Approximately 70% of cases of chlamydia are asymptomatic [2]. Because of its asymptomatic nature, many at risk individuals do not go for chlamydia testing. Countries have begun responding to this understanding by introducing screening programs that can proactively detect and treat the infection.

There are two principle types of chlamydia screening program. *Population screening *involves offering all eligible members of a population a screening test. *Opportunistic screening *involves offering tests to eligible people while they are attending a service setting for reasons that are usually unrelated to their sexual health. 'Eligible' individuals are those whose personal demographic profiles match the demographic profile of the population that is being targeted by the screening program. For example if a screening program is attempting to reach young women between the ages of eighteen and twenty-nine years of age, any young woman in this age range who comes in contact with the program would be deemed 'eligible'. The focus of this paper is on opportunistic screening. Though the rationale for screening for chlamydia appears to meet Wilson & Junger's [3] criteria, there has recently been some controversy over or not whether opportunistic screening on its own can reduce the prevalence of *Chlamydia trachomatis*- see Low, [4]. However opportunistic approaches provide important supplementary supports to more systematic population screening efforts, and therefore understanding how individuals respond to opportunistic screening is important.

Much of the work that has been completed on opportunistic screening for chlamydia has concentrated on the technical aspects of screening, such as calculating infection rates. Though such research is important, what is often omitted by it are young people's views on screening. Though there have been some exceptions [5,6], researchers have generally highlighted the limited nature of the work that exists on what young women think and feel about screening. The limited work on this topic suggests that young people may find it acceptable to be offered screening [6], though a number of factors (such as stigma) may reduce acceptance rates [7]. Knowledge about *why *young people choose to accept screening offers is also limited [7].

Drawing on semi-structured qualitative interviews with thirty five young women, this article examines the following questions:

• How would young women in Ireland feel about being offered opportunistic chlamydia screening, and what factors would encourage these young women to accept opportunistic testing?

• What are the best ways to offer young women opportunistic tests for chlamydia, and who should do this?

• What factors influence young women's partner notification preferences (who would they notify in the event that they tested positive for chlamydia as a result of taking part in a screening program)?

## Methods

Ethical approval for the study was received from our institutional ethics committees. We utilised a qualitative approach because we wanted to explore young people's perspectives in detail.

Participants were recruited from six GP settings and two female family planning clinics. These settings were chosen because they would be the types of settings that would offer opportunistic screening to adolescents and young adults if a national screening program were to be introduced in Ireland; getting the perspectives of young adults from these settings was therefore a priority. Receptionists in these settings were asked to hand out recruitment leaflets to all of their young adult patients (both males and females). These leaflets stated that young adults who were interested in taking part in the study should text 'yes' to the lead author's mobile phone number and that each interviewee would be provided with a thirty euro gift voucher for their time. At the end of a two-week intensive receptionist-led recruitment period recruited forty-one potential respondents were recruited (forty one women and two men). Four of these women subsequently dropped out of the study, and we decided to exclude the two male participants from the study in order to focus on the perspectives of the young women. We therefore ended up interviewing thirty-five young women. It is important to note that when we initially designed this study we did not intend to focus exclusively on the perspectives of young women; this was a decision that was forced upon us by our inability to recruit young men (we theorise about why young men might have been reluctant to take part in this study in the limitations section of the discussion).

Six respondents were recruited from urban family planning clinics, eight from an urban GP practice situated in a working class area, five from a student GP setting, seven from two rural GP settings and another final seven respondents from two urban GP practices located in middle class areas. We did not explicitly target heterosexual women, and did not ask interviewees to delineate their sexuality; all respondents made reference to either currently having boyfriends, or having had sex with men in the past. Eight respondents were in their late teens, five were in their early 20s, seven were in their mid-20s and fifteen were in their late 20s.

The first named author (male) carried out 30 interviews and a public health specialist (female) who had received training in qualitative data collection conducted five (average length of interviews: one hour). Similar themes arose in both sets of interviews, indicating that the interviewers' gender did not positively or negatively impact the quality of the data that was generated in the interviews. Prior to this study, the first author had conducted similar qualitative studies with young women with chronic (diabetes) and developmental (Asperger syndrome) conditions.

A standard open-ended approach to questioning was used. Interview questions examined: how respondents thought they would feel if they were offered chlamydia testing; what concerns, if any, they would have about chlamydia testing; how they thought screening offers could best be made to them; and who they thought would be the best person to make them these offers. They were also asked about their (partner) notification preferences. We felt that we reached data saturation (the point where we felt no new salient themes were emerging in the interviews) around interview twenty five, but we continued interviewing until we had interviewed the entire sample of thirty-five women.

Respondents were given the option of completing their interviews either by telephone or face-to-face. Eighteen respondents completed interviews over the telephone and seventeen face-to-face. Telephone interviews were useful for us as they maximised cost-effectiveness and convenience. Some respondents preferred telephone interviews because they felt that these interviews afforded them more privacy and confidentiality than they would have had during a face-to-face interviews. Similar themes came up in both the face-to-face and the telephone interviews. Respondents who completed face-to face interviews were asked to provide written consent to be interviewed; respondents who completed telephone interviews were asked to supply verbal consent.

Respondents were not offered chlamydia screening as part of this study, and were not screened for chlamydia prior to being invited to take part in it.

A medical secretary transcribed the interviews as they were completed. All words in the interviews were transcribed but not all para-linguistic expressions such as pauses. Analysis did not begin until all of the interviews had been transcribed. During the first phase of analysis significant key words, phrases and themes were marked in the transcripts with summary words or codes that labeled them for subsequent analysis. As each transcript was coded, all codes that were thematically similar were grouped together, and labeled with a summary code, called a category. As categories were developed we began to develop an explanatory framework that could be used to explain how different categories related to each other. Each interview was read and coded by the first, second and third authors. The fourth and fifth authors then provided feedback on these codes, and on emerging framework that these codes supported.

## Results

Results are summarised in five main sections: a). screening as a threat to young women's identities; b). subgroups of young women who were especially (un)concerned about screening; c). why young women would choose to accept screening; d). how young women wanted to be notified of negative test results; e). and young women's partner notification preferences. In line with standard qualitative approaches, discussion is interwoven the results.

### Screening as an identity threat

Most respondents indicated to us that they would react negatively (at least initially, and possibly only momentarily) to an unexpected chlamydia screening offer.

I'd be kind of shocked [if I was offered a test]. (Female no. 1, late teens, rural GP).

I'd feel ashamed. (Female no. 17, late 20s, urban middle class GP).

Respondents indicated that routine medical encounters (encounters between patients and healthcare professionals that were undertaken for any purpose apart from sexual advice giving/help-seeking) were interactions where it was important to maintain identities as 'normal' young women. Normalcy for these young people had sexual connotations: it meant appearing to be 'good girls' rather than 'bad girls', that is appearing to be sexually responsible individuals who were not promiscuous. Respondents enacted 'good girl' identities in medical encounters by carefully regulating their verbal and non-verbal behaviors and the topics that they addressed in the interaction (i.e. avoiding topics and behaviours that would signify promiscuity). By engaging in these practices respondents were able to appear to be 'good girls', which in turn enabled them to claim positive social value for themselves and avoid information emerging during the medical encounter that would discredit their identities.

Respondents also (implicitly) believed that a specific interaction-script governed the encounter between healthcare professional and patient. This script stipulated that healthcare professionals would provide healthcare advice in a way that would not discredit or undermine patients' identities.

Three features, then, characterised the medical encounter between respondent and professional from respondents' perspectives: the need for respondents to maintain a 'good girl' identity, the expectation that the healthcare professional would not threaten this identity and the assumption that the medical encounter would be governed by a script that directed the behavior of the actors involved in it along fairly conventional lines.

Respondents felt that opportunistic testing could challenge all three of these features. The interaction script that respondents had prepared themselves for- and expected medical professionals to take - no longer applied i.e. a healthcare professional could ask a respondent if she wished to take a test without the respondent having asked for it first; confusion could result as respondents struggled to understand the deviation from the expected interaction script.

I suppose at the initial suggestion there'd always be a little feeling of shock, a bit of 'oh God, where did that come from, we were here talking about my hand or whatever!' (Female no. 23, mid 20s, rural GP).

If all of a sudden the doctor says, 'oh, can we check you for chlamydia', it's going to knock you for six. You're not expecting to talk about that! (Female no. 22, late teens, student GP).

The offer itself could threaten respondents' sense of themselves as 'good girls' by implying that they were promiscuous risk-takers who were 'in need' of STI testing. Respondents often had negative images of the types of individuals whom they thought would require chlamydia testing (often referring to these women as 'sluts', 'skanks' and 'slags'); they strongly associated STIs such as chlamydia with deviant female subcultures and identities [8]. Respondents ideas about STIs here drew on common representations of STIs as 'symbls of moral corruption', associations of STIs with promiscuity, dirtiness and low class status [8]. Respondents felt that screening offers could have the potential to forcibly equate them with women in these negative identity categories, and could lead them, if they were offered screening, to experience scary and stigmatizing redefinions of their social and sexual selves [8]. Nack [8] notes that having learned to associate STIs with promiscuity, young women often perceive STI-related diagnostic encounters to be unexpected and threatening. This is because STIs are incongrous with the master standards of femininity and feminine goodness to which young women often attempt to subscribe [8]. Respondents were especially anxious about the thought of being offered screening on a risk factor basis, where healthcare professionals would make judgements that respondents should be offered screening because their sexual behaviours deviated significantly from the norm, i.e. because they possessed attributes that clearly differentiated them from other good, responsible women [8].

Interviewer: Why do you think people would be offended if someone brought it [Chlamydia testing] up with them?

Respondent: It's just that you're insinuating something about this person. You're almost criticizing them, saying that they're a certain type of person (Female no. 31, late 20s, rural GP).

Interviewer: So if you knew that you were being offered screening because the doctor thought that you were being more risky [than other young women] you'd be a bit more offended?

Respondent: I would feel offended if I was singled out for testing. That seems ridiculous but I think I would honestly. It would be important to say that everyone's being tested. It would make it more normal, to say everyone's doing it. It's a bit more acceptable. (Female no. 13, late teens, student health GP).

### Groups especially (un)concerned about the screening offer

Younger respondents and those recruited from working class and rural settings had the strongest screening-related identity concerns. These respondents were embedded within compact social networks that were characterised by high levels of peer surveillance. Relative to their older counterparts and their counterparts recruited from middle-class GP settings these respondents described more community monitoring of their sexual and health and service seeking practices and a concomitant greater need to appear to be 'good girls'. These respondents felt that their identities could easily and permanently become damaged if individuals in their social networks discovered that they had engaged in 'discrediting activities', such as accepting screening. Hyde and Howlett [9] note that young working class women often experience greater policing of their sexual practices than young people from middle-class areas do; they also note that young women from working class backgrounds tend to be more sexually unassertive than their middle-class counterparts and more likely to consent to their sexual practices being judged and policed. Respondents in this study who were from working class and rural areas also feared that the clinic staff who would offer them screening, or the reception staff who would process their results, would personally know respondents' family members or friends. If this was the case these healthcare professionals could (in)advertently leak discrediting information about respondents to these individuals.

Coming from a small town where everybody knows each other, you'd probably be terrified that it would get out [that had accepted screening offer] (Female no. 6, mid 20s, urban middle-class GP).

If someone found out the whole place would know, everyone. Everybody would know. You know yourself, Chinese whispers. (Female no.9, late 20s, urban working-class GP).

If someone in college read a text message on your phone that you'd taken a test it get around the whole class. It'd be like, 'oh my god, do you know what she has, you'll never guess'. It would be just how people could see you. Everybody's looking going 'I wonder what she was at'. They would get a bad negative picture of you. (Female no. 13, late teens, student health GP).

Older respondents and those recruited from middle class urban settings generally expressed less intense concerns about screening. These respondents trusted that healthcare professionals would not leak discrediting information about them to other people (often because the healthcare professional did not know their friends of families). It may also have been the case that older respondents were embedded within less dense, and therefore less surveillant social networks, than their younger counterparts, who may have lived with their parents and have been in school or college.

I don't see why you'd be so worried about it. The doctors not going to tell anyone else as you've got doctor-patient confidentiality. (Female no.34, late 20s, student health GP).

I'd find it ok to be offered a test. If you were on the pill you'd have to have the smear test done every so often. I wouldn't feel uncomfortable doing it. (Female no. 23, mid 20s, rural GP).

### Characteristics of the healthcare professional offering the opportunistic test

Respondents indicated that they would not accept screening from 'para-professionals' (healthcare professionals who were neither doctors nor nurses) who worked in public settings (e.g. public pharmacies, clinic reception areas).

Interviewer: How would you feel about [accepting a test from] a receptionist?

Respondent: No way. No way. Everyone would be looking at you (Female no.23, mid 20s, rural GP).

Respondents were happy to be offered screening by both doctors and nurses.

A doctor, not a nurse. (Female no.34, late 20s, student health GP).

A nurse, as long as it was in private. (Female no.7, late 20s, urban middle-class GP).

Most respondents wanted to be offered screening by younger female healthcare professionals.

Interviewer: Is there anything that would discourage you from taking the test - either in how it's being asked or...

Respondent: As long as it'd be a female your own age that would be dealing with you (Female no. 23, mid 20s, rural GP).

Respondents felt that younger female professionals would be more likely to empathise with them and to understand their identity related concerns, since these professionals (presumably) experienced similar concerns in their own everyday lives.

I just think it's a lot easier to talk to a woman when there's something wrong. Especially about women's stuff. They'd understand more. (Female no. 18, late teens, rural GP).

You can connect with someone the same sex. (Female no.28, early 20s, urban working class GP).

Respondents were less enthusiastic about being offered screening by either older female health professionals or by males. Respondents believed that older individuals would have more conservative attitudes about sexuality than would younger people, and that men would be more judgmental about women's sexuality than would other women. Consequently these professionals would lack protective orientations towards respondents' identies during medical encounters. Respondents 'good girl' identities would therefore be at great risk of being discredited during interactions with these professionals. Overall, respondents expressed a dislike for what Nack [10] refers to as 'moral surveillance' models of doctor-patient interaction. Nack [10] notes that healthcare workers who employ moral surveillance interaction styles with patients during medical encounters often exercise their power and authority in such a way that they communicate negative judgements about patients to patients. The result is that patients often go away from those encounters feeling that they are depraved, promiscuous deviants [10]. In contrast, respondents expressed preferences for healthcare workers who employed patient-centred models of interaction [10]. These are models of interaction where healthcare workers are alive to patients' emotional well-being and identity concerns as well as they are to their physical health. Respondents' accounts indicate that they believed that younger female healthcare workers would be more likely to employ patient-centred interaction models, and males and older females would be more likely to employ moral surveillance methods.

The doctor we have is young and is very open and she sort of knows everything. She's not like, you know like older people are a bit ... they look down on you but she doesn't. I think men...just automatically assume that you're sleeping around rather than thinking there's other ways of catching [STIs]. Just women are more understanding so it would be better with a female doctor. (Female no.33, late 20s, urban middle-class GP).

Men doctors would just look at you like you were a little slut or something. (Female no.35, late 20s, urban working-class GP).

I don't think girls would do it cos if the doctors were male and I think they'd be a bit embarrassed to take the test with their doctor. (Female no.35, late 20s, urban working-class GP).

Respondents indicated that they would use a variety of strategies to assess how empathetic individual doctors or nurses would be to their identity related concerns, including paying close attention to healthcare professionals' non-verbal expressions and tone of voice. Screening offers put forward by healthcare professionals deemed to be judgemental would likely be refused.

Respondent: I'd listen to them while they're offering. You can tell it [that they're judging] by the sound of their voice.

Interviewer: And what can you actually tell from the sound of the voice?

R: I don't know. You just hear something different in their voice. There's just a sort of cold tone to their voice (Female no.35, late 20s, urban working-class GP).

How screening offers were framed was also thought to be important. Offers that were framed in such a way that they employed moral surveillance styles of interaction and attacked and undermined respondents' 'good girl' identites were likely to be rejected; offers that supported these identities, or at least did not threaten them, were more likely to be accepted.

Ask me have I any concerns about my own health and stuff like that. Say things like 'for your own safety, you'd be better off taking a test? Don't say anything that would make someone feel dirty. (Female no.21, late 20s, urban working-class GP).

### Screening as a support for moral identites

Despite the concerns that respondents raised about screening, most indicated that they would accept screening if it was offered to them.

I've no problems in relation to going about doing it (Female no.24, early 20s, urban middle-class GP).

Respondents gave a number of specific reasons for accepting screening, including concerns about their future reproductive health and concerns about previous risky sexual activities (such as having sex with strangers without using condoms). The most common reason for accepting screening, however, was that screening was seen as a good thing to do: it was viewed as a moral practice.

I think once a year every person should really have this test done. You know. Just to make sure that they are clear and alright. (Female no. 23, mid 20s, rural GP)

I think that's a good practice to get screened. (Female no.28, early 20s, urban working class GP).

Increasingly in what Giddens [11] refers to as 'late modern' societies (Western post-industrial consumer societies) individuals are expected to take control of their own health, and to desire to improve it [11]. Emphasis is placed in these societies on self-regulation and self-control (and being seen to be self-regulating and self-controlling), to the extent that individuals may be stigmatised if they are thought of as being 'careless' or 'irresponsible' with their health [12]. Accepting an STI screening offer would enable respondents to identify with this healthist discourse; it would allow them to feel pride in the adequacy of their self-identities and practices and enable them to signal to healthcare professionals and peers that they were individuals who could look after their own health and bodies.

If you're starting off a relationship with someone and you could say 'I've been tested I know I have nothing'. It would be a lovely thing to be able to say to somebody. You'd feel good about yourself if you could say that to someone else. (Female no. 2, late 20s, family planning).

If you accept screening you're looking after yourself and respecting your body. I think it's been instilled in me from a young age that if you respect your body, other people will respect your body too. (Female no. 26, late 20s, family planning).

Morality was also implicated in another reason that two respondents gave for accepting screening: the need to protect screening professionals' identities. These respondents felt that healthcare professionals could themselves be embarrassed if respondents were to reject their offers. Accepting screening would prevent this embarrassment from occurring and therefore help professionals' to maintain their appearances of competence.

It wouldn't be so bad if I went voluntarily myself whereas if you're there yourself and you're asked you feel you have to do. You wouldn't want to embarrass them or yourself. (Female no.5, late teens, student health GP).

Respondents were more likely to accept screening if healthcare professionals emphasised to them that screening was a normal practice that many young people engaged in and stressed that testing was a good, responsible thing to do. The consequences of such practices would be to reduce the threat that screening posed to respondents' good girl identities while maximizing the supporting effects that screening had for respondents' healthy identities.

Well I think if they were to do an awareness campaign, not to single out, to use everybody from a certain age. Don't break it down to individual people. Whereas if it's a wider age group it's not as bad. It doesn't appear as bad. (Female no. 25, late teens, student health GP).

If you just put it [screening] to them [young adults] it's just for their health and it'll be good for them. Don't make them feel ashamed or dirty about it. We're not saying it because you're sleeping around with loads of people. (Female no.9, late 20s, urban working-class GP).

If I knew loads of people did accept it and get it done I would. I'm very much about what other people think. (Female no.6, late teens, urban middle-class GP).

Most respondents who indicated that they would accept screening did not believe that they were at risk of having chlamydia. Several respondents noted that they would be disinclined to accept screening if they believed that they might have chlamydia. For individuals with a perceived low risk of having chlamydia, screening supported their healthy identities without threatening their good girl images. For individuals with a perceived high risk of having chlamydia, screening threatened their good girl images without necessarily having benefits for their healthy identities. The stigma of having an STI was such that it could outweight the benefits that came from engaging in a healthy activity such as testing.

Interviewer: So for what reasons would you decide to do it [accept test] if someone asked you?

Respondent: I don't know, just to be curious really. Just, you know, like that I wouldn't have known what happened previous to my partner. I wouldn't know who he's ever been with previous to myself.

I: Do you feel that you might be at risk now?

R: No. (Female no.33, late 20s, urban middle-class GP).

Interviewer: Is there anything that you think would put somebody off having the test if they were offered it?

Respondent: Well if they felt, they knew that they could have something that would deter them from wanting to have it done. (Female no. 31, late 20s, rural GP).

### Notification preferences

Respondents were asked about how they would like to be notified of their test results if they tested negative for chlamydia. Respondents were given four methods by which a negative diagnosis could be communicated to them and asked to pick which method they would prefer to be notified by, and why. These methods were: by email, by text message, by phone call or by letter. Most respondents wanted to be notified by mobile (cell) phone or text message. The principle benefit of this method of notification would be information control: stigmatising information about respondents would be exchanged directly between doctor and respondent so there would be little risk of other people finding out about it [13]. Other methods of notification (letter/email) were riskier because other individuals (over whom respondents had no control) could monitor the information flow between professionals and respondents.

Well, all the IT [information technology] guys in work have access to our email accounts. I just wouldn't like to take the risk. So just calling my mobile would be best really (Female no. 2, late 20s, family planning).

You wouldn't want your parents finding out [that you had taken a test] so you wouldn't want a letter turning up on the door and your mam saying what's that for? So I suppose a text or a call (Female no.7, late 20s, urban middle-class GP).

### Moral aspects of partner notification

Respondents were asked about their partner notification preferences. All respondents indicated that they would inform their current partners themselves if they tested positive for chlamydia. About two thirds of respondents indicated that they would inform their previous partners.

Respondents had a number of concerns about informing their partners (both current and previous), however. Informing partners that respondents had been diagnosed with chlamydia could undermine the positive 'good girl' images that respondents had established for these partners. Respondents were also worried about the risk that their previous partners would inform other people that respondents had tested positive for chlamydia.

Interviewer: What would be the most worst thing about telling the ex partners?

Respondent: Just to be stigmatised about sleeping around. They'd probably think you were sleeping with everybody. (Female no.21, late 20s, urban working-class GP).

If you were going out with someone do you really want to tell them you've got an STD. It's the way other people will look at them (female no. 3, late teens, student health GP).

Because of these concerns several respondents indicated that they would engage in aggressive impression management activities with their partners if they tested positive. This meant that respondents would preemptively accuse their partners of infecting them with chlamydia so as to deflect the negative identity consequences of having an STI from themselves. This finding echoes Nack's [14] work on 'stigma transference'. Nack found that young women who had been diagnosed with HPV often blamed their partners in light on a postive STI diagnosis so as to preserve their images as good girls. Aggressive impression management would be particularly important in situations where knowledge of respondents' diagnoses had become public knowledge. At this point respondents' good girl images would be discredited and a limited number of face-saving actions would be available to them. The most appealing strategy to take in such a situation would be to act like a victim, even though this line could only be taken as a result of destroying their partners' social identity.

Respondent: If I caught something the first thing I'd be doing is running up and saying 'what did you give me?' That's the first thing I'd do. I'd blame him straight away. It would just take the heat off you I suppose!

Interviewer: What do you mean by the heat?

R: Well, for him to turn around and call you names, you know saying well who were you with, you must have been with this, that and the other. So I'd be blaming him, let him worry about it, you know what I mean? It's just so you wouldn't get all the slack. (Female no.35, late 20s, urban working-class GP).

Why, then, would most respondents notify their partners in light of the concerns expressed in these narratives? Respondents saw notification as the right thing to do, as a moral duty. Respondents, who were female, were particularly concerned about the harmful effects that chlamydia could have on their partners' past or future girlfriends.

You couldn't not tell them. It would be bad karma. It just wouldn't be good, wouldn't be fair. (Female no. 6, mid 20s, urban middle-class GP).

I suppose the guilt thing would not be that I passed it onto him but it might get passed on to a future partner of his. (Female no. 23, mid 20s, rural GP).

I just think morally it would be incorrect not to tell people. (Female no. 3, late teens, student health GP).

## Discussion

This article has examined how young women would respond to an offer of a chlamydia test in a primary care setting. Though there has been previous research on this topic, most of it has been conducted with young people recruited from urban STI or family planning clinics [15]. Our study focuses on the perspectives of young people recruited from community-healthcare settings, and from both urban and rural regions. A particular strength of out work is that it unpicks differences between young women from urban and rural areas and from middle-class and working-class backgrounds in relation to how these young women would respond to opportunistic chlamydia screening offers.

### Situating the findings in relation to previous empirical research on Chlamydia screening

We found that screening could pose a number of challenges for our respondents. It could disrupt the taken-for-granted nature of the nurse/doctor-patient interaction that they expected to experience during the medical encounter. It could also threaten what we (drawing on the work of authors such as Nack [8]) refer to as respondents' 'good girl' identities, i.e. their need to maintain sexually respectable appearances in front of other people, including healthcare professionals. Respondents felt that their social positions as 'good girls' could be undermined if they were to accept screening (see La Rusch et al. [16] for more about young women's anxieties about these issues). In effect, they indicated that they would interpret the screening offer in light of prediagnostic social lessons that they had learned about the differences between 'bad' girls and 'good' girls [8]. Previous studies of young women's feelings about chlamydia and STI screening in the UK, the U.S. and Australia have found anxieties about promiscuity and identity discreditation that are similar to the ones identified here [17-20]. Our findings also indicate, however, that young Irish women are not homogeneous in terms of their performance-related preferences and that 'good girl' performance concerns in the context of chlamydia screening are likely to be strongest amongst young Irish women from lower socio-economic backgrounds and younger women. This finding correlates with La Rusch et al's [16] work which show that young women with lower levels of education (who are likely to be from lower class backgrounds) are likely to experience greater levels of perceived STI-related stigma than their more educated counterparts.

Most respondents wanted to be be offered screening by young, female healthcare professionals, feeling that these professionals would be best placed to understand their identity and stigma related concerns. In Nack's (2008) terms respondents wanted to be offered screning by healthcare workers who would employ patient-centred rather than moral surveillance methods of interaction. Preferences were also expressed for being offered screening in private areas, rather than in more public settings (for example, reception areas), again presumably because these areas had greater identity support effects than more exposed settings. Similar preferences have been expressed in previous studies [21]. We believe that this is the first study to note (albeit in relation to a small minority of respondents, n = 2) that young adults' identity related concerns in the opportunistic screening encounter extend to their fellow interactant, the healthcare professional; that one of the reasons why a young person might accept screening would be to protect the identity of the screening professional.

Despite the potentially upsetting effects of chlamydia screening, however, most respondents indicated that they would accept chlamydia screening if it was offered to them. Screening enabled respondents to feel that they were healthy individuals who took personal care of their bodies and of their health. Similar findings have been found in other studies [22], though this is the first study to note that the health benefits that young adults associate with chlamydia screening themselves have an impression management dimension; in their narratives respondents sometimes imagined themselves revealing their (negative) results to other individuals, the purpose of which would be to show to these people that respondents were healthy, positive individuals who were deserving of respect.

Respondents expressed concerns about notifying their partners about their screening activities. International studies support our finding that young women often feel anxious about informing their partners about their STI testing activities [14]. These studies also support our finding that young women are often more concerned about informing their previous sexual partners about positive STI diagnoses than they are about informing their current ones.

### Study limitations

We cannot say for sure if there were any gaps in the recruitment of young women for this study. Though we asked reception staff to hand out recruitment leaflets to all of their patients, this might not have happened. Other studies show that recruiters for sexual health studies often do not approach all eligible people due to lack of knowledge about screening, worries about discussing sexual health issues and a lack of guidance [23]. There was also a fairly high dropout rate amongst the young women who agreed to be interviewed. Several factors may have discouraged the young women who dropped out from completing interviews, including anxiety about being interviewed by a male interviewer, the interview material being too sensitive [24], not being recompensed enough for taking part in an interview, and time pressures. The data presented in this study is drawn from a self-selected sample of young women; other young women may have greater or lesser concerns about chlamydia screening.

Secondly, we managed to only capture the perspectives of young women. This is an important absence. Men are just as likely to have and to spread chlamydia as young women and ignoring their perspectives means that we are not addressing an important vector of the chlamydia pathogen. Concentrating on women also, perhaps, helps to reinforce the stereotypical assumption that sexual health is a 'woman's issue'. International research suggests that there are a number of reasons why young men might decline to take part in a study such as this one. One is lack of knowledge about chlamydia; men often do not know very much about chlamydia or why it is important, which can disincentivise them from taking part in STI-related research studies [25]. Men also have stronger feelings of STI-related invulnerability than women, which may lead them to think STIs (and by implication STI-related research studies) are irrelevant to them [26]. From a more pragmatic perspective, time and money concerns may have prevented young men from taking part in the study. It is possible that if we increased the incentive for taking part in the study from thirty euros to fifty or more, more men would have wished to take part.

The thid limitation of the study is the hypothetical nature of its results. The women who took part in this study were not offered chlamydia screening and so their accounts must be interpreted carefully. What this article presents is data relating to what women say they would do about chlamydia screeening, not data about what they would actually do; respondents' narratives and concerns may have little relation to their practices. Other studies support the utility of the kind of information collected by this study, however. These studies suggest that while individuals' intentions to act in certain way (such as intending to notify partners in the event of a positive diagnosis of chlamydia) often do not have linear causal impacts on their behavior, they do have important influences [27]. However, it is important to acknowledge that other factors such as perceived self-efficacy, habits and subjective norms can also influence individuals' behaviours [27]. On the positive side, where screening is offered to young women the uptake appears to be fairly good [28].

A fourth limitation is that we did not ask female respondents to delineate their sexual orientation. This may be an important absence because young women's screning preferences may be correlated with their sexual orientation/identities. It would be useful therefore for future research studies to examine whether or not sexual orientation is a variable that influences young Irish women's screening preferences.

## Conclusion

The clearest message coming through from this study is that chlamydia screening, treatment and partner notification needs to be normalised and destigmatised if they are to be made acceptable to young women. This finding is consistent with previous research in this area. Healthcare workers making screening offers to young women should stress to them that screening is being offered to all young women and not just a few promiscous deviants. Healthcare professionals also need to monitor their own interaction styles with patients. Professionals who use moral surveillance methods of interacting with patients should be considered unsuitable for engaging in the kind of work described in this article, or receive feedback on how they could change their interaction approach so as to make it more suitable to patients. Professionals should frame the screening offer to emphasise the health benefits of screening. Partner notification support should be offered to patients who test positive for chlamydia. Healthcare workers should offer contact tracing where young people are reluctant to inform their previous partners. Using the strategies described here would address the concerns raised by young women in this study, and help to ensure the greatest population coverage of chlamydia screening.

## Competing interests

The authors declare that they have no competing interests.

## Authors' contributions

MB carried out thirty of the interviews, analyzed all of the interviews and drafted the manuscript (apart from the methods section). RB designed the study and analyzed all of the interviews. EOC carried out five of the interviews and analyzed all of the interviews. DOD designed the study and analyzed all of the interviews. DV analyzed all of the interviews and and drafted the methods section of the paper. All of the authors read and approved the final manuscript.

## Pre-publication history

The pre-publication history for this paper can be accessed here:

http://www.biomedcentral.com/1471-2458/10/425/prepub
